# Case Report: Solid variant of papillary thyroid carcinoma in a young adult with Turner syndrome with chronic thyroiditis

**DOI:** 10.3389/fonc.2023.1150002

**Published:** 2023-11-09

**Authors:** Daichi Murakami, Masayoshi Hijiya, Takuro Iyo, Sachiko Hayata, Takashi Ozaki, Keisuke Enomoto, Masamitsu Kono, Shunji Tamagawa, Muneki Hotomi

**Affiliations:** ^1^ Department of Otorhinolaryngology Head and Neck Surgery, Kinan Hospital, Wakayama, Japan; ^2^ Department of Otorhinolaryngology Head and Neck Surgery, Wakayama Medical University, Wakayama, Japan; ^3^ Department of Pathology and Clinical laboratory, Kinan Hospital, Wakayama, Japan

**Keywords:** solid variant of papillary thyroid carcinoma, Turner syndrome, chronic thyroiditis, gene mutation, gene panel test

## Abstract

Turner syndrome is associated with an increased risk of developing several neoplasms. In particular, a clinical feature of Turner syndrome with chronic thyroiditis implies a relationship with thyroid malignancies. We report a very rare case of a solid variant of papillary thyroid carcinoma that was identified during a follow-up of chronic thyroiditis in a 22-year-old woman with Turner syndrome. The patient had no notable history of radiation exposure. No genetic mutations relating to the occurrence of the solid variant of papillary thyroid carcinoma, including *RET*/PTC rearrangements and mutations in the *BRAF* or *RAS*, were detected by a gene panel test, namely, the Oncomine™ Dx Target test. To the best of our knowledge, this is the first report of a solid variant of papillary thyroid carcinoma in a young adult with Turner syndrome with chronic thyroiditis. Our case suggests that in patients with Turner syndrome, there may be different pathogeneses from those previously reported, including exposure to radiation or known genetic mutations for the development of a solid variant of papillary thyroid carcinoma.

## 1 Introduction

Turner syndrome (TS) is a common chromosomal abnormality resulting from structural or numeric abnormalities in the X chromosome (1, 2). Individuals with TS are at an increased risk of developing multiple neoplasms (3–5). Approximately 1.3 - 4.8% of patients with TS have some kind of neoplasms (5, 6). Patients with TS have a two- to five-fold increased risk of benign central nervous system (CNS) tumors, colorectal cancers, and skin cancer (6). Moreover, some studies have reported the association between TS and thyroid malignancy (1, 3–5, 7, 8). Notably, however, all previous reports have focused on conventional papillary thyroid carcinoma (PTC). A solid variant of papillary thyroid carcinoma (SVPTC) is a rare form of PTC, accounting for just 1−3% of all PTC subtypes. SVPTC displays a neoplastic cell growth with a solid and syncytial pattern surrounded by a thin fibrous stroma (9–11). The clinical course of SVPTC is still controversial. Some studies, such as those by Vural C et al. and Vuong HG et al., showed that SVPTC had a higher risk of recurrence or distant metastases than classical PTC. The authors listed SVPTC as an aggressive variant (10, 12). However, the study by Ohashi R concluded that it was difficult to strictly determine the prognosis of SVPTC because of the ambiguous histological diagnostic criteria and the lack of information on the genetic background (11). These indicate the importance of accumulating the clinical cases of SVPTC, including genetic backgrounds. SVPTC can occur in patients exposed to high levels of radiation, and cases were reported, for example, in association with the 1986 Chernobyl nuclear accident (13, 14).

Recently, however, there have been reports of SVPTC without a notable history of radiation exposure, and the pathogenesis has attracted a lot of attention (9, 10, 12, 13, 15). Gene mutations could be involved in the development of SVPTC. Previously, *RET*/PTC rearrangements and mutations in the *BRAF* and *RAS* were reported to be related to SVPTC (9–11, 13–17). Accumulation of further genetic pathogenesis of SVPTC is required. Besides, chronic thyroiditis, linked with an increased risk of development of differentiated thyroid carcinoma (7, 18–20), could be associated with SVPTC without a notable history of exposure to radiation (9, 10). TS is characterized as an X chromosomal abnormality and is often accompanied by chronic thyroiditis (2, 3). Considering these clinical features, TS may lead to the development of SVPTC without previous incidence of notable irradiation.

Herein, we report the first known case of SVPTC in a young adult with TS with chronic thyroiditis. We attempted to elucidate the genetic background of SVPTC in TS in this patient using the Oncomine™ Dx Target test (Life Technologies Corporation, Carlsbad, CA). Characteristics of TS including genetic background and chronic thyroiditis were suggested to be related to the development of SVPTC. Written informed consent for this study was obtained from the patient herself.

## 2 Case description

**Figure 1:**
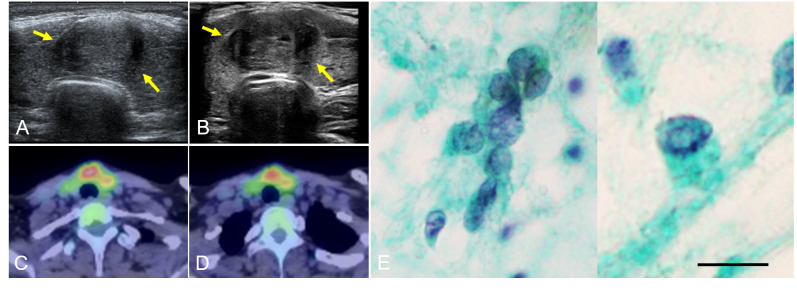
Thyroid ultrasonography examination showed a tumor increasing in size over a 19-month period, from 19.7 × 12.9 × 19.4 mm **(A)** to 22.0 × 12.7 × 27.6 mm at the time of presentation **(B)**. Arrows show the tumor extent. PET-CT showed a tumor with strong FDG accumulation (SUV max: 8.36) **(C)** and another part of the thyroid with diffuse FDG accumulation **(D)**. Fine needle aspiration cytology showed ground glass nuclei and nuclear pseudoinclusion (intranuclear cytoplasmic inclusion) **(E)** (Papanicolaou staining. Scale bar is 20 μm). PET, positive emission tomography; CT, computed tomography; FDG, fluorodeoxyglucose; SUV, standardized uptake value.

A 22-year-old woman with TS presented a thyroid tumor located within the isthmus close to the left thyroid lobe, which was seen to be increasing in size during ultrasound thyroid monitoring of chronic thyroiditis. In her childhood, the patient’s pediatrician regularly performed thyroid function tests to check thyroid dysfunction associated with TS. At the age of 14 years, she was noted to have hypothyroidism and the diagnosis of chronic thyroiditis was reached because of the elevation of anti-thyroglobulin antibody and anti-thyroid peroxidase antibody. She had been taking levothyroxine at 25 µg/day as a treatment for a mild form of thyroid hormone deficiency from chronic thyroiditis. She had a history of treatment with somatropin of growth hormone (GH) at a dose of 0.35mg/kg/week between the ages of 4 and 18 years. During treatment with GH, insulin-like growth factor (IGF) 1 level was routinely monitored and had been at normal range from 6 to 8 years old. When the endocrinologist took over her follow-up from pediatricians at the age of 19 years, the endocrinologist performed the first thyroid ultrasound examination and detected the thyroid tumor. It was 1 year after the completion of GH treatment and 3 years prior to the presentation to our department. There was no notable history of radiation exposure. She had no family history relating to thyroid tumors or thyroid dysfunction. Blood exams revealed anemia and increasing levels of transaminase, thyroglobulin, anti-thyroglobulin antibody, and anti-thyroid peroxidase antibody (**Table 1**). Other thyroid functions were in the normal range. At the first thyroid ultrasound examination 3 years prior to the current presentation, the initial diameters of the tumor were 18 x 12 x 18 mm. The diameters of the tumor almost did not change, however, at the current presentation, ultrasound showed a tumor increasing in size from 19.7 × 12.9 × 19.4 mm at 19 months previously to 22.0 × 12.7 × 27.6 mm (**Figures 1A, B**). Enhanced computed tomography (CT) and positron emission tomography (PET)-CT showed a tumor of the left thyroid lobe with lymph node swelling in the central compartment of the neck. The tumor of the left thyroid lobe showed strong fluorodeoxyglucose (FDG) accumulation (standardized uptake value (SUV) max: 8.36). In contrast, the thyroid showed diffuse FDG accumulation, suggesting chronic thyroiditis (**Figures 1C, D**). The result of fine needle aspiration cytology was “Suspicious for Malignancy” based on the Bethesda system. Cytology showed the signs of papillary thyroid carcinoma, such as ground glass nuclei and nuclear pseudoinclusion. Because there were not enough cell clusters to make a definitive diagnosis of papillary thyroid carcinoma, the pathologist made a diagnosis of “suspicion of papillary thyroid carcinoma” (**Figure 1E**). Although two enlarged lymph node swellings in the lateral compartment of the neck were also detected by preoperative ultrasound examination and PET-CT, fine needle aspiration cytology revealed that both of them were reactive lymph nodes and without malignancies. Under the diagnosis of cT2N0M0 and classification into an intermediate-risk group, we performed subtotal thyroidectomy including the isthmus and left lobe and central neck dissection, based on clinical practice guidelines for Thyroid Tumors by the Japan Associations of Endocrine Surgeons (21). There were no complications or recurrence of nerve injury or hypoparathyroidism after the surgery.

**Table 1:** Blood test.

Parameter	Result	Unit
WBC	5,800	/µL
Hb	9.8	g/dL
PLT	21.4	x 10^4^/µL
Total protein	7.5	g/dL
Albumin	3.7	g/dL
ALT (GOT)	117	IU/L
AST (GPT)	134	IU/L
LD	186	IU/L
Creatinine	0.41	mg/dL
Glucose	96	mg/dL
CRP	0.69	mg/dL
Na	137	mEq/L
K	4.1	mEq/L
Cl	101	mEq/L
Ca	8.9	mg/dL
TSH	4.013	µU/mL
FT4	0.89	ng/dL
Tg	120.7	ng/mL
Tg-Ab	9.76	IU/mL
TPO-Ab	477.5	IU/mL
TSHR-Ab	1.9	IU/mL

WBC, white blood cells; Hb, hemoglobin; PLT, platelets; ALT, alanine aminotransferase; GOT, glutamic oxaloacetic transaminase; AST, aspartate aminotransferase; GPT, glutamic pyruvic transaminase; LD, lactate dehydrogenase; CRP, C-reactive protein; TSH, thyroid stimulating hormone; FT4, free thyroxine; Tg, thyroglobulin; Tg-Ab, anti-thyroglobulin antibodies; TPO-Ab, anti-thyroid peroxidase antibodies; TSHR-Ab, anti-thyroid stimulating hormone receptor antibodies.

**Figure 2:**
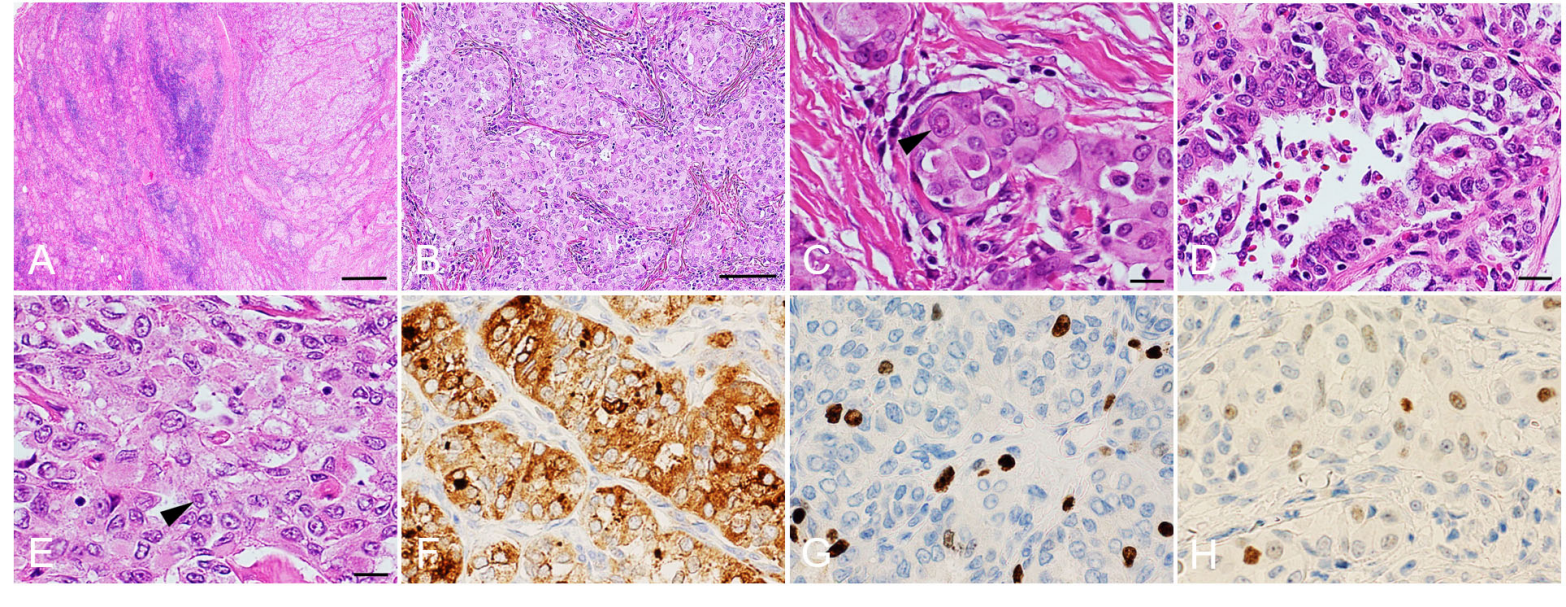
Histopathological analysis. Hematoxylin and eosin staining **(A–E)**. Papillary thyroid carcinoma on the right side and chronic thyroiditis on the left side **(A)**. Solid and trabecular patterns within the carcinoma **(B)**. The arrowhead shows a nuclear pseudoinclusion (intranuclear cytoplasmic inclusion) **(C)**. Nuclear grooves are slightly conspicuous in carcinoma cells **(D)**. Ground glass nuclei are conspicuous in carcinoma cells and nuclear pseudoinclusions are partly identified (arrowhead) **(E)**. Immunohistochemical analyses show expression of thyroglobulin **(F)**, MIB-1 (MIB-1 labeling index: 5−10%) **(G)** and P53 **(H)**. Scale bars: 400 μm **(A)**, 100 μm **(B)** and 20 μm **(C–H)**.

Histopathology showed that the tumor consisted of a solid-trabecular structure of multiple vascular and lymphatic invasions with chronic thyroiditis (**Figures 2A, B**). The tumor had the characteristics of PTC with nuclear pseudoinclusion (intranuclear cytoplasmic inclusion) (**Figure 2C**) and nuclear grooves (**Figure 2D**). Ground glass nuclei were conspicuous in carcinoma cells and part nuclear pseudoinclusions were partly identified (**Figure 2E**). Based on immunohistochemical staining, there was a diffuse expression of thyroglobulin in the tumor cells (**Figure 2F**). The tumor cells expressed MIB-1 with a labeling index of 5-10% (**Figure 2G**). P53 was partially expressed in the tumor cells (**Figure 2H**). There were lymph metastases in the central compartment of the neck. The histopathological diagnosis was SVPTC (pT2N1a) with chronic thyroiditis. Gene mutational analysis using the Oncomine™ Dx Target test (approved by the Kinan Hospital Ethical Review Board, approval number: 245) revealed that the patient did not show certain genetic mutations including *RET*/PTC, *BRAFV^600E^*, *RAS*, *PIK3CA*, *AKT1*, or *NTRK*. At the time of this report, the absence of recurrence and metastases was assessed by regular post-operative ultrasonography. In addition, PET-CT at 12 months after the surgery showed no signs of recurrence or metastasis.

## 3 Discussion

SVPTC occurred in a young adult patient with TS associated with chronic thyroiditis. Gene mutation analysis showed that she had none of the previously reported gene mutations, so there is thought to be a different mechanism behind SVPTC specific to TS. This could be chronic thyroiditis or a genetic background differing from those previously reported.

Chronic thyroiditis associated with TS might, for example, be related to the development of SVPTC. SVPTC is a rare variant of PTC and the incidence is associated with exposure to certain types of radiation (13). The patients of PTC with TS in our study and previous studies were young, with ages ranging from 11 to 40 years, and some patients were described to be associated with chronic thyroiditis or to have a history of treatment with GH (1, 7, 8, 22). Chronic thyroiditis, reported in 24−55% of patients with TS (7, 23), is reported to be associated with SVPTC (9, 10, 15) or PTC displaying prominent stromal desmoplasia and marked fibrosis (18, 24, 25), which are the pathological characteristics of SVPTC. In previous studies, thyroid nodules were detected in 13−18.8% of patients in the ultrasound follow-up of chronic thyroiditis, and PTC was observed in 10.3−22.5% of those with nodules (19, 20, 26). Chronic thyroiditis has been suggested to be related to thyroid malignancy, including PTC, but the exact mechanism remains unclear (5). Related to endocrine disorders, some patients with PTC with TS have a history of GH treatment (1, 7, 8), including the patient of the present study. GH treatment has been reported to be related to the occurrence of several malignancies, although the correlation between GH treatment and PTC is controversial (1, 7, 8, 27). Although it was unclear if there was radiation exposure in some patients in the previous studies, the similarities in endocrine circumstances between our and previous studies would support the mechanism of PTC with TS during younger age and be the basis of the development of SVPTC associated with TS.

Genetic background resulting in TS might also be a factor in the development of SVPTC. The frequency of SVPTCs carrying genetic mutations is considered to be different from that of ordinary PTCs, 70% of which are reported to have genetic mutations (11). Among patients with SVPTC who had not been exposed to radiation, some gene mutations, such as *RET*/PTC rearrangements (9–11, 13–16) and mutations in *BRAF* ^V600E^ or *RAS* (11, 17), were said to be related to the pathogenesis of SVPTC. *RET*/PTC rearrangement is the most common gene mutation among SVPTC and is detected in 43% of cases of SVPTC (13). Mutations in the *BRAF* ^V600E^ and *RAS* are the next most common mutations, detected in 11% and 39% of cases, respectively (17). Genetic alterations related to the activating mitogen-activated protein kinase (MAPK) signaling pathway, *RET/PTC* rearrangements, and mutations in *BRAF^V600E^* or *RAS* are reported to be present in SVPTC. In addition, other activating mutations such as *PIK3CA* or *AKT1* due to genetic alterations in the PI3K/Akt signaling pathway are also involved (28, 29). *NTRK* fusions are other genetic alterations reported to be relatively rare in PTC but more common in advanced thyroid carcinoma (15, 28, 29). Our patient had no gene mutations according to the Oncomine™ Dx Target test. However, in previous studies, the genetic background of PTC associated with TS had not been discussed except in the study of Papanikolaou et al. (22). As for SVPTC in patients with TS, this is the first study discussing genetic background. Because *RET*/PTC rearrangements and *BRAF* and *RAS* gene mutations occur exclusively with each other in SVPTCs, the estimated incidence of SVPTC without genetic mutation is only 7%. In addition, an increasing number of studies have shown a higher incidence of other genetic mutations such as *NTRK* (30). While there have been no studies covering genetic mutations in SVPTC, it is presumed that SVPTCs without genetic mutations are very rare. Regarding SVPTC development in patients with TS, there might be different genetic pathogeneses from those reported, such as X-chromosome gene haploinsufficiency (3). Some studies have demonstrated the association between chronic thyroiditis and X-chromosome gene haploinsufficiency in TS. Impairment of forkhead box P3 (FOXP3), which locates on the X-chromosome and acts as both activator and repressor of regulatory T cells (Tregs), causes the suppression of Treg function and autoimmune thyroiditis (31). FOXP3 also influences tumor immunity. In breast cancer, loss of nuclear FOXP3 results in overexpression of HER2, a member of the transmembrane receptor tyrosine kinases, and activation of the downstream MAPK and PI3K/Akt signaling pathway (32, 33). In ovarian cancer, upregulation of FOXP3 decreases the expression of Ki-67 (34). Impairment of FOXP3 by haploinsufficiency could conversely cause the increase of Ki-67. Lower expression of Ki-67 (MIB-1labering index) will be important for the diagnosis of SVPTC. Although not all of the pathogenesis of SVPTC in TS patients can be explained, FOXP3 would be an attractive molecule for future studies and investigations. Recently, genetic mutations affecting other than the abovementioned signaling pathways, such as *p53*, *Wnt/β-catenin*, and *TERT* promoter mutations, have been reported in all histological types of thyroid cancer type (29). However, these mutations were not excluded in the present study. Accumulation of cases and future studies on these genetic backgrounds are needed.

In conclusion, a rare variant SVPTC occurred in a young adult patient with TS with chronic thyroiditis. In patients with TS, there might be different pathogeneses from those previously reported. Exposure to certain radiation or particular genetic mutations could be involved in the development of SVPTC. This case provides some evidence for neoplasm in patients with TS, but future studies are required to elucidate the genetic mechanism.

## 4 Patient perspective

I was diagnosed with Turner syndrome as an infant and had ultrasound thyroid monitoring of chronic thyroiditis. Solid variant papillary thyroid carcinoma was diagnosed. The surgeon recommended gene mutation analysis. I was worried that I would find a genetic mutation with a bad prognosis, but the analysis was inconclusive. Fortunately, there have been no signs of recurrence or metastasis since the surgery.

## Data availability statement

The datasets presented in this article are not readily available because of ethical and privacy restrictions. Requests to access the datasets should be directed to the corresponding author.

## Ethics statement

The studies involving humans were approved by the Kinan Hospital Ethical Review Board. The studies were conducted in accordance with the local legislation and institutional requirements. The participants provided their written informed consent to participate in this study. Written informed consent was obtained from the individual(s) for the publication of any potentially identifiable images or data included in this article.

## Author contributions

DM conceptualized and wrote the first draft of the manuscript. DM, MHi, TI, and SH managed the patient and critically reviewed the manuscript. TO provided information about the histopathology of the case. KE, MK, and ST reviewed and edited the manuscript. MHo supervised the writing process and editing during the manuscript writing process. All authors contributed to the article and approved the submitted version.
